# THE RELATIONSHIP BETWEEN PERSONAL AGING ANXIETY AND ATTITUDES TO OLDER PATIENTS AMONG HEALTHCARE PROFESSIONALS

**DOI:** 10.1093/geroni/igz038.313

**Published:** 2019-11-08

**Authors:** Jenny Inker

**Affiliations:** Virginia Commonwealth University, Richmond, Virginia, United States

## Abstract

Nearly 20% of adults age 50 and older report experiencing age discrimination by a healthcare professional. Numerous harmful health consequences flow from ageism in healthcare encounters, including failure to accurately diagnose and respond to treatable conditions such as pain and depression. Despite this, little is known about ageism among healthcare professionals, including whether job role and work setting influences attitudes to older patients. This study aims to use relational ageism theory to explore the relationship between personal aging anxiety among healthcare professionals and their attitudes to older patients, including potential moderating factors of job role and work setting. A convenience sample of healthcare professionals working for a small regional health system in the US (N = 145) completed an online survey using the Aging Anxiety Scale to measure personal aging anxiety and the Geriatric Attitudes Scale to measure negative attitudes to older patients. Regression analyses demonstrated that personal aging anxiety significantly predicted attitudes to older patients, with greater anxiety associated with more negative attitudes. Job role significantly predicted attitudes to older patients with physicians’ attitudes being more negative compared to other disciplines, while work setting was not predictive of attitudes toward older patients. This research confirms the need to provide ageism awareness training for healthcare professionals and to include in this training an exploration of internalized attitudes to aging. Further research with a larger, more representative sample is indicated to test replication and to better understand how to overcome job role risks of ageism among healthcare professionals.

